# An uncommon cause of seizures in children living in developed countries: neurocysticercosis -a case report

**DOI:** 10.1186/1824-7288-37-9

**Published:** 2011-01-25

**Authors:** Irene Raffaldi, Carlo Scolfaro, Federica Mignone, Sonia Aguzzi, Federica Denegri, Pier-Angelo Tovo

**Affiliations:** 1 Department of Pediatrics, Division of Infectious Diseases, University of Turin, Regina Margherita Children's Hospital, Piazza Polonia 94, 10126, Turin, Italy; 2 Department of Neuroradiology, Orthopaedics and Traumatology Hospital, Via Zuretti 29, 10126, Turin, Italy

## Abstract

Neurocysticercosis represents an important cause of seizures in children in endemic countries, such as Latin America, Asia and sub-Saharan Africa, while in Europe, especially in Italy, the cases of neurocysticercosis are anectodal. We report the case of a 6 year old boy, born and lived for four years in Cameroon, who presented a right emiconvulsion. The diagnosis was neurocysticercosis. This case accentuates the need to consider neurocysticercosis in a child presenting with non febrile seizures, mainly if he emigrated from an area of high prevalence or if he had long-term stay in endemic regions.

## Background

Neurocysticercosis represents an important cause of seizures in children in endemic countries. It is due to the brain involvement by the larval stage of the cestode Taenia solium (cysticerci). This parasite is commonly found in developing countries of Latin America, Asia and sub-Saharan Africa [1-5]. The prevalence of neurocysticercosis in some of these countries exceeds 10% [6]; conversely in Europe the cases of neurocysticercosis are anecdotal, especially caused by migratory flows from endemic zones or international travels. The case reported below describes a rare cause of seizures in a child who lives in a developed country.

## Case presentation

### Case report

A 6 year old boy who was born and lived in Cameroon for four years, in a rural area, was admitted to the Emergency Department with seizure lasting more than thirty minutes, not responsive to Diazepam 0.5 mg/Kg e.r. The patient had immigrated to Italy two years previously. No familiarity for seizures or headache. At the age of 1 year the child had experienced an acute and isolated febrile seizure with oculogyration. He had no recent history of traumatic or infective episodes, neither ingestion of drugs or weight loss. When he woke up that morning, he had speaking difficulties, right deviation of the mouth, followed by right head and gaze deviation, right emiclonic convulsion, and loss of consciousness. At admission the child was afebrile, unconscious, with accelerated heart rate and respiratory difficulties. His head was deviated to the right, there was a generalized hypertonia with hyperextension of right arm and flexion of the left arm. The electroencephalogram (EEG) detected a slow mono-polymorphic activity on the left and central electroencephalographic leads. This activity was absent on the right leads. It suggested a post-critical focal cerebral suffering.

**Figure 1:**
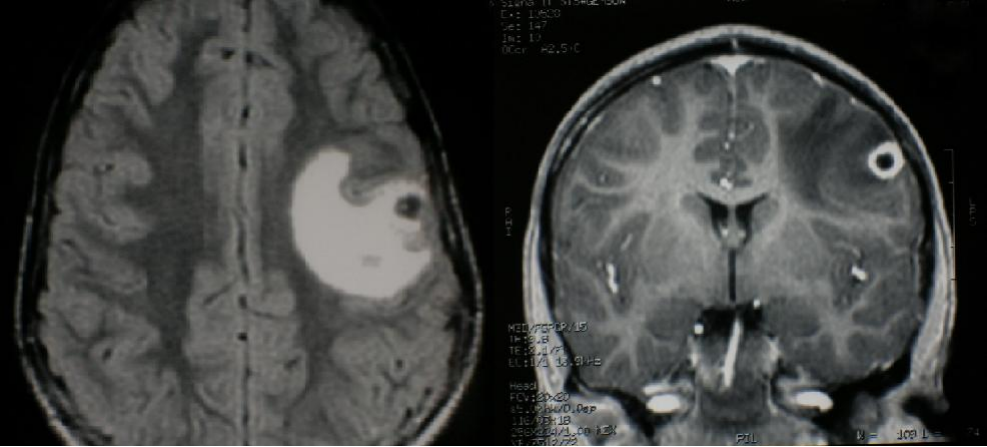
**Brain MRI before specific treatment**. (a) axial T2-weighted MRI shows an alteration in left frontal-parietal cortex attributing to vasogenic edema (b) coronal T1-weighted post contrast MRI shows a little ring-enhancing lesion in left frontal cortex.

He was directly admitted to the Intensive Care Unit and he was successfully treated with Phenobarbital (5 mg/Kg i.v.). The magnetic resonance imaging (MRI) of the brain showed two cystic round lesions located one in the right lentiform nucleus and one in the left frontal-parietal lobe (Figure 1). In the following 36 hours the EEG displayed an improvement of child's cerebral conditions with the disappearance of the asymmetric slow activity reported previously. After 48 hours the child was transferred to our Department. Blood exams revealed high eosinophil cell count (720 cells/μL) and Ig E levels (217 UI/ml). Western blot assay detected specific antibodies against cysticercus (LDBIO DIAGNOSTICS, Lyon, France). Therefore the diagnosis of neurocysticercosis was made and the appropriated therapy was started: the child received, orally, albendazole (15 mg/Kg/day) in two divided doses and dexamethasone (2 mg/day) for eight days.

**Figure 2:**
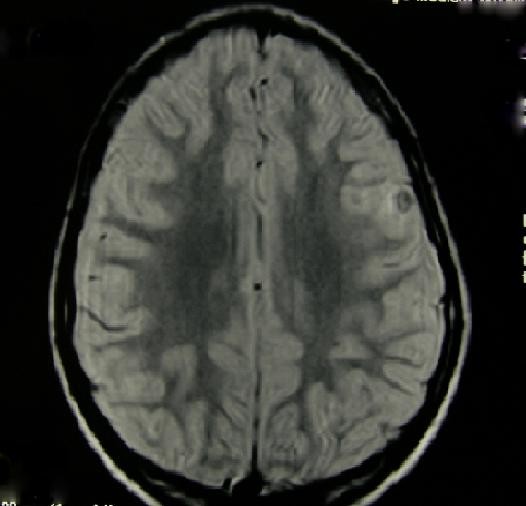
**Post treatment brain MRI**. Axial T2-weighted MRI shows edema's disappearance and calcified lesions, one in right lentiform nucleus and one in left frontal-parietal cortex.

Six months later, the eosinophil and IgE levels got back to normal. At brain MRI calcified areas, corresponding to the previous lesions, were indicated, without any new active lesions (Figure 2).

In the following two years the patient remained seizure-free on oral antiepileptic therapy. Then the patient has been lost to follow up.

## Discussion

Neurocysticercosis is the most common parasitic infection of central nervous system [7]. Taenia solium, responsible of the disease, can causes, in individuals, intestinal infection and cysticercosis. In the classic life cycle humans develop taeniasis after eating not well cooked pork meat, containing cysticerci. Larvae develop into adult tapeworm inside human's small intestine. The tapeworm releases daily eggs disseminated to the environment through feces. In developing countries, where sanitary regulations are often neglected, roaming pigs may ingest food polluted by eggs, becoming the intermediated host. Once in the pig's intestinal tract, eggs embrionate into oncospheres, which cross the intestinal wall and, through bloodstream, migrate to the target tissues (brain, muscle, subcutaneous surface). The cycle can be interrupted if individuals accidentally ingest eggs through food or water contaminated by feces. In this case, the parasite perceives the human as an intermediate host and disseminates to a variety of organs, including the central nervous system, where cysticerci develops [8].

The clinical manifestations of neurocysticercosis are variable, depending on the number, stage, location as well as the host response [9,10]. A large part of individuals holding the parasite in the central nervous system are asymptomatic [8]. In some patients symptoms may develop many years after the brain infection, as it has happened to our patient. The most frequent manifestations, due to parenchymal location, are seizures, learning disability, difficulty with balance, behavior changes. Extraparenchymal location of the parasite causes increased intracranial pressure with headache, vomiting and papilloedema [7,8,11].

Parenchymal forms of neurocysticercosis have a better prognosis than extraparenchymal forms, in term of remission of clinical signs [7]. Carpio and Hauser noticed that the risk of seizure recurrence is high after a first acute symptomatic fit but the risk seems related to the persistence of active brain lesions [12].

Histological demonstration of parasites from a brain biopsy should be the safe "gold standard" for the diagnosis of neurocysticercosis. Clearly this procedure is limited because of its invasiveness. Therefore neuroimaging remains the main instrument for the diagnosis. In the identification of extraparenchimal cysts MRI is more sensitive than Computed Tomography (CT) scan [10].

Immunological tests have low sensitivity and specificity, especially for single lesion. The enzyme-linked immunosorbent assay has 65% specificity and 50% sensitivity and it can give false negative results in case of parenchymal neurocysticercosis, inactive lesion or helminthic infections. The enzyme-linked immunoelectrotransfer blot assay, using purified glycoprotein antigens from Taenia solium cysticerci, has been reported to be highly specific (100%) and nearly 98% sensitive for patients with either multiple active parenchymal cysts or extraparenchymal neurocysticercosis [10,13].

The differential diagnoses include echinococcosis, pyogenic brain abscess, fungal abscess, tubercoloma, toxoplasmosis, a primary or metastatic tumor and infectious vasculitis [14].

Antiparasitic drugs are the mainstay of treatment; in particular, albendazole may be favourable for the treatment of parenchymal cysts because of its power to pass into cerebral spinal fluid [8]. Previous studies recommended the administration of albendazole at a dosage of 15 mg/Kg/die for 1 month, but later studies prove that a 1 week course is equally effective [14]. Corticosteroids are useful for reducing local edema and inflammation around dying parenchimal cysts; so they are often administered together with antiparasitic drugs. Kalra displayed that the association of albendazole and dexamethasone increases complete or partial resolution of lesions and reduces the risk of recurrence of seizure among children presenting one or two ring-enhancing lesion on CT [15].

This case is interesting mainly for epidemiological reasons. In fact neurocysticercosis is endemic in Latin America, Africa and some Asiatic countries. In Europe, many cases have been reported in Portugal, Spain, Poland and Romania. In Italy it is a rare disease. In recent years no cases have been described, but with high rate of immigration from endemic areas (Africa and East Europe) this parasitosis will be found in our country too [16].

## Conclusions

This case emphasizes the need to consider a parasitic infection of the central nervous system, particularly neurocysticercosis, in a child with an onset of epilepsy especially if he had long-term stay in endemic regions, or he emigrated from an endemic area, although not recently. In case of relevant suspicion of neurocysticercosis it needs testing serum cysticercal antibody and making neuroimaging examinations.

## Consent

Verbal informed consent was obtained from the patient's parents for publication of this case report and any accompanying images, at time of diagnosis; then the patient (with his family) has been lost to follow up.

## Competing interests

The authors declare that they have no competing interests.

## Authors' contributions

IR, CS, FM, SA have made useful contribution in drafting the manuscript and in the revision of the literature.

PAT has been involved in revising it critically for important intellectual content. FD has participated in the diagnostic pathways. All authors read and approved the final manuscript.

## References

[B1] Roman G Sotelo J Del Brutto O Flisser A Dumas M Wadia N Botero D Cruz M Garcia H De Bittencourt PRM Trelles l Arriagada C Lorenzana P Nash TE Spina-Franca A A proposal to declare neurocysticercosis an international reportable disease Bull World Health Organ 2000 78 3 399 406 10812740 PMC2560715

[B2] Grill J Rakotomalala W Andriantsimahavandy A Boisier P Guyon P Roux J Esterre P High prevalence of serological markers of cysticercosis among epileptic Malagasy children Ann Trop Paediatr 1996 16 185 191 8893946 10.1080/02724936.1996.11747824

[B3] Singhi S Singhi P Clinical profile and etiology of partial seizures in North Indian infants and children J Epilepsy 1997 10 32 36 10.1016/S0896-6974(96)00073-4

[B4] Gaffo AL Guillén-Pinto D Campos-Olazábal P Burneo JG Cysticercosis as the main cause of partial seizures in children in Peru Rev Neurol 2004 39 10 924 926 15573306

[B5] García HH Gonzalez AE Evans CA Gilman RH Taenia solium cysticercosis Lancet 2003 362 9383 Cysticercosis Working Group in Peru 547 556 12932389 10.1016/S0140-6736(03)14117-7 PMC3103219

[B6] Ong S Talan DA Moran GJ Mower W Newdow M Tsang VC Pinner RW EMERGEncy ID NET Study Group Neurocysticercosis in radiographically imaged seizure patients in U.S. Emergency Departments Emerg Infect Dis 2002 8 6 608 613 12023918 10.3201/eid0806.010377 PMC2738481

[B7] Carpio A Neurocysticercosis: an update Lancet Infect Dis 2002 2 12 751 762 10.1016/S1473-3099(02)00454-1 12467692

[B8] Mody R Nield LS Stauffer W Kamat D Seizure in a 20-month-old native of Minnesota: a case of neurocysticercosis Pediatr Emerg Care 2005 21 12 860 862 10.1097/01.pec.0000190232.20233.45 16340766

[B9] Garcia HH Del Brutto OH Cysticercosis Working Group in Peru Neurocysticercosis: updated concepts about an old disease Lancet Neurol 2005 4 10 653 661 10.1016/S1474-4422(05)70194-0 16168934

[B10] Singhi P Singhi S Neurocysticercosis in children J Child Neurol 2004 19 7 482 492 10.1177/08830738040190070201 15526951

[B11] San-Juan Orta D Clinical manifestations of neurocysticercosis Neurologia 2009 24 5 331 335 19642036

[B12] Carpio A Hauser WA Prognosis for seizure recurrence in patients with newly diagnosed neurocysticercosis Neurology 2002 59 11 1730 1734 12473760 10.1212/01.wnl.0000036320.69823.ea

[B13] Ramos-Kuri M Montoya RM Padilla A Govezensky T Diaz ML Sciutto E Sotelo J Larrade C Immunodiagnosis of neurocysticercosis. Disappointing performance of serology (enzyme-linked immunosorbent assay) in an unbiased sample of neurological patients Arch Neurol 1992 49 6 633 636 1596199 10.1001/archneur.1992.00530300069012

[B14] Sinha S Sharma BS Neurocysticercosis: a review of current status and management J Clin Neurosci 2009 16 7 867 876 10.1016/j.jocn.2008.10.030 19394828

[B15] Kalra V Dua T Kumar V Efficacy of albendazole and short-course dexamethasone treatment in children with 1 or 2 ring-enhancing lesions of neurocysticercosis: a randomized controlled trial J Pediatr 2003 143 1 111 114 10.1016/S0022-3476(03)00211-7 12915835

[B16] Carangelo B Erra S Del Basso De Caro ML Bucciero A Vizioli L Panagiotopoulos K Cerillo A Neurocysticercosis. Case report J Neurosurg Sci 2001 45 1 43 46 11466507

